# Correction: Angiotensin-Converting Enzyme (ACE) 2 Overexpression Ameliorates Glomerular Injury in a Rat Model of Diabetic Nephropathy: A Comparison with ACE Inhibition

**DOI:** 10.1186/s10020-022-00482-9

**Published:** 2022-05-04

**Authors:** Chun Xi Liu, Qin Hu, Yan Wang, Wei Zhang, Zhi Yong Ma, Jin Bo Feng, Rong Wang, Xu Ping Wang, Bo Dong, Fei Gao, Ming Xiang Zhang, Yun Zhang

**Affiliations:** 1 Key Laboratory of Cardiovascular Remodeling and Function Research, Chinese Ministry of Education and Chinese Ministry of Health, Shandong University Qilu Hospital, Jinan, Shandong China; 2 Cellular Immunology Laboratory, School of Medicine, Tsinghua University, Beijing, China

## Correction: Molecular Medicine 17, (2011) 59-69 10.2119/molmed.2010.00111

Following publication of the original article [1], the authors found an error in Fig. 1B: the immunohistochemical images in the ACEI and AD-ACE2 + ACEI groups were mistakenly used, which were partially overlapped with the correct image in the Ad-ACE2 group. They went back to their raw data and found the original immunohistochemical images in the ACEI and AD-ACE2 + ACEI groups (n = 10 in each group). The corrected Fig. 1B is given in this correction article.

**Fig. 1:**
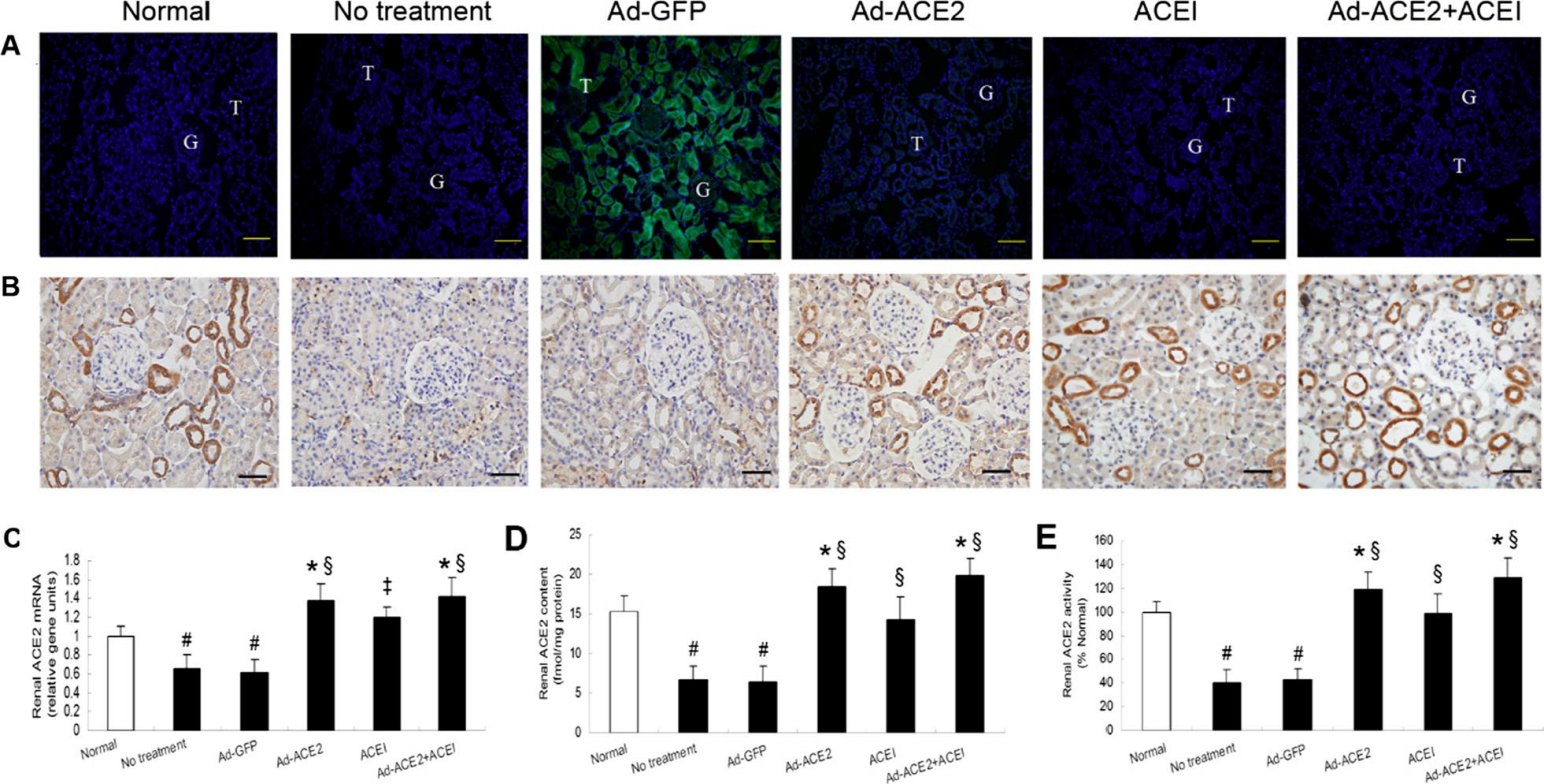
Efficiency of gene transfer 4 wks after gene delivery. **A** Fluorescence microscopic images of frozen sections of the kidneys in the normal, no treatment, Ad-GFP, Ad-ACE2, ACE1 and Ad-ACE2 + ACEI groups, respectively (bar = 50 μm). G indicates glomeruli and T indicates tubules. **B** Immunochemical staining showing the localization of ACE2 protein in cortical sections from each group. Weak ACE2 staining was observed in tubules from rats in the no treatment and Ad-GFP groups, whereas marked ACE2 staining was depicted in both tubules and glomeruli from rats in the normal, Ad-ACE2, ACEI and Ad-ACE2 + ACEI groups (bar = 50 μm). **C, D, E** Quantitative analysis of ACE2 mRNA levels, protein levels and activities. *P < 0.05, ^#^P < 0.01 versus normal group; ^‡^P < 0.05, ^§^P < 0.01 versus no treatment group

The authors apologise for this error.
